# Editorial: Digital health and neuroscience: Recent history, current trends, and future developments

**DOI:** 10.3389/fnint.2022.1073682

**Published:** 2022-11-14

**Authors:** Miguel Pais-Vieira, Arjun Ramakrishnan, Eduardo Rocon, Mikhail Lebedev, Odete A. B. da Cruz-e-Silva

**Affiliations:** ^1^Department of Medical Sciences, Institute of Biomedicine (iBiMED), University of Aveiro, Aveiro, Portugal; ^2^Department of Biological Sciences and Bioengineering, Indian Institute of Technology Kanpur, Kanpur, India; ^3^Centre for Automation and Robotics, Technical University of Madrid, Spanish National Research Council, Madrid, Spain; ^4^Vladimir Zelman Center for Neurobiology and Brain Rehabilitation, Skolkovo Institute of Science and Technology, Moscow, Russia

**Keywords:** mobile health, health information technology, wearable devices, telehealth, telemedicine, personalized medicine

## Introduction

The topic “*Digital Health and Neuroscience: Recent History, Current Trends, and Future Developments*” aims to summarize current perspectives, present new findings, and discuss opinions and proposals on the past, present, and future developments between neuroscience and digital health. Establishing these associations is important to determine to what extent neuroscience can benefit from advances in digital health and *vice-versa*.

**Figure 1:**
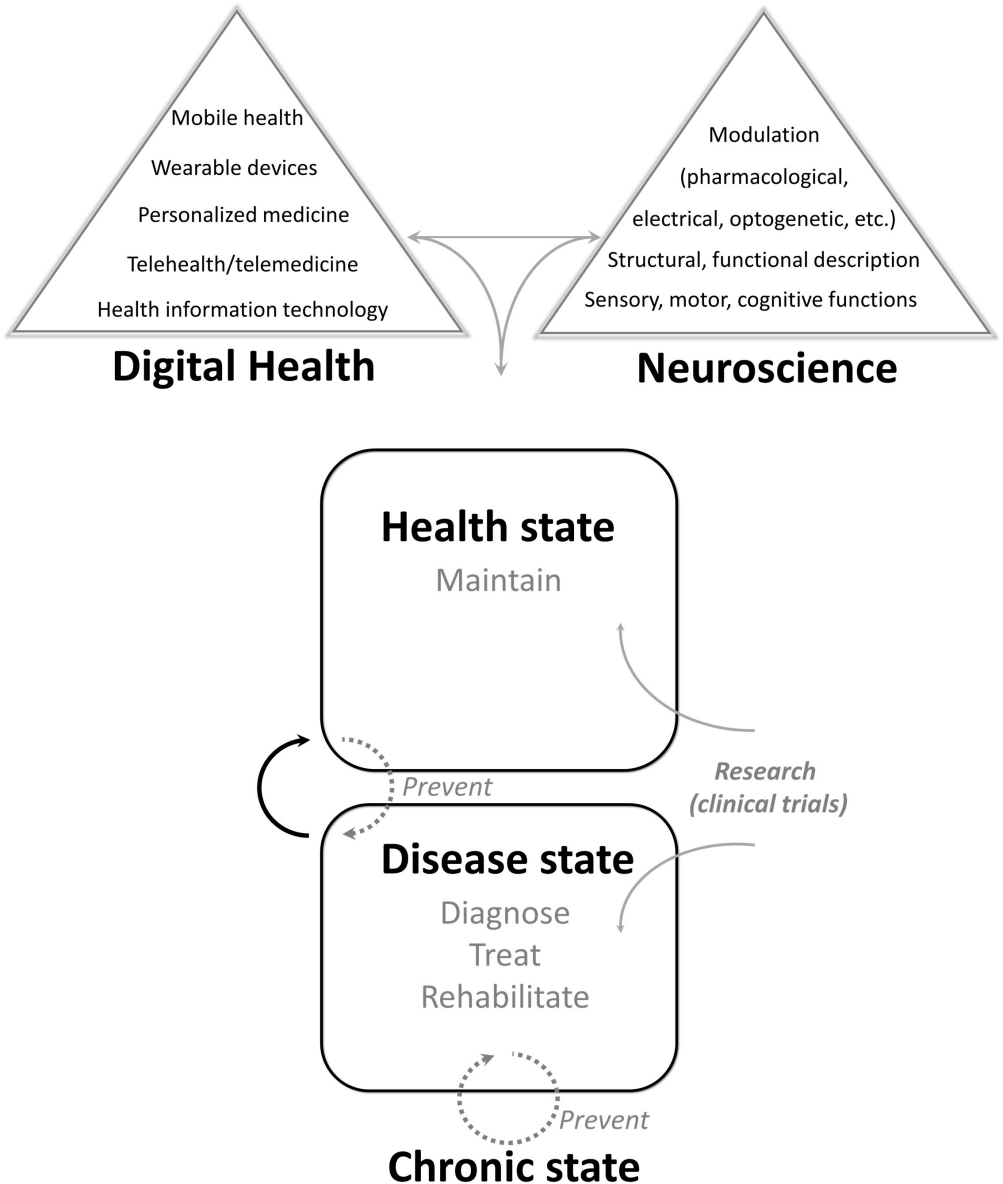
Digital health and neuroscience.

The techniques and themes of the studies composing this Research Topic are indicative of the research avenues that are being pursued within the field of digital health and neuroscience. According to the Food and Drug Administration (FDA)^1^, digital health is composed of the subfields of mobile health, health information technology, wearable devices, telehealth/telemedicine, and personalized medicine (Figure 1). In this Research Topic, four of the five studies could be included in the subfield of information technology, three in the subfield of precision medicine, and one in telemedicine. Regarding the pathologies studied, two of these studies were related to stroke, another two to Alzheimer's disease, and the last one consisted of a neuropsychological assessment, with relevance to both stroke and Alzheimer's disease.

The use of machine learning to improve ischemic stroke diagnosis or identify risk factors was the subject of two studies. The study by Maharjan et al., which can be included in the subfield of health information as well as in precision medicine, uses machine learning to identify the best candidates to perform clinical trials related to ischemic stroke. More specifically, the authors study health records using a gradient-boosted machine learning algorithm (MLA) to predict which patients were at risk of developing an ischemic stroke within a 1-year period. Although other studies have used algorithms to predict the onset of ischemic stroke, they are typically oriented toward longer time periods. However, to make decisions on the best candidates for clinical trials, it is critical to be able to predict which individuals will have a stroke within the study period. The study by Xu et al., is also a retrospective study in the subfield of health information and precision medicine. The authors use different machine learning algorithms to predict complications in ischemic stroke, more specifically hemorrhage transformation. The authors find that the random forest model presented the best predictions. In addition to the comparison of the sensitivity and specificity of the algorithm (i.e., report true positives and false negatives), these authors also report the SHapley Additive exPlanation values. These values, which are used to eliminate the need for artificial intelligence and machine learning as a black box, suggest that triglyceride, hemoglobin, and Lipoprotein (a) levels, as well as the results from the National Institutes of Health Stroke Scale, are the main factors affecting the random forest model predictions. These two retrospective studies demonstrate how machine learning is being used to improve patient selection for clinical trials as well as to predict complications.

Two other studies used machine learning to improve current methods to diagnose Alzheimer's disease. Namely, Zhang et al. develop and validate a sensitive and selective immunoassay for total tau in plasma. For this, they combine the analysis of total tau protein in plasma, with other clinical variables and use different machine learning algorithms to identify the most relevant clinical features. Using this information, they demonstrate that a nomogram including the tau level and the clinical features allows for the best predictions. Meanwhile, Lu et al. also use machine learning to improve the diagnosis of Alzheimer's disease. However, they have done so through the analysis of functional magnetic resonance activity. More specifically, they propose a Key Features Screening Method based on Extreme Learning Machine (KFS-ELM) that uses high-level features. The authors report that different ELMs are sensitive to different features, and in addition, all these features are important for Alzheimer's disease classification. Therefore, they combine all these features in their classifier and find a positive correlation between the number of features and the accuracy of the classifier. These are another two examples of health information and precision medicine studies but, in this instance, aimed at diagnosing Alzheimer's disease, thus combining basic neuroscience with machine learning.

Lastly, the study by Arioli et al. is a validation of the Remote Characterization Module that allows testing multiple standardized neuropsychological measures. The authors compare the results of tests when older subjects were in a remote location (i.e., at home) with in-person paper-and-pencil tests. This approach is of particular interest not only because it allows for remote testing, but also because it is based on a text-to-speech application programming interface (API), which allows the user to speak instead of having to write or use a keyboard. The authors show that this module permits testing verbal long-term as well as working memory, verbal fluency, and set-shifting. The results are comparable to population-based age-normalized scores. This study constitutes a good example of the challenges faced by health professionals in the implementation of remote evaluation but is also a good example of the advantages of telemedicine/telehealth. Of note, the authors report that despite the use of a video connection that allowed the user to communicate with, and visualize the tester, the results of some remote tests may have been influenced by lower attention levels. Meanwhile, given the recent context of the COVID-19 pandemic, the ability to perform a remote neuropsychological evaluation of multiple cognitive domains in older people constitutes a significant step toward a generalized use of telemedicine/telehealth in diagnosis and follow-up.

Taken together, the collection of manuscripts in the present Research Topic, suggests that digital health, and especially the subfields of health information and precision medicine, can largely benefit from the potential of combining machine learning with existing neuroscience techniques to maintain health, and improve diagnosis, treatment, and follow up. Although the present collection supports a fundamental role for machine learning in health information technology and personalized medicine, it is likely that two-way dialogue between neuroscience and digital health will continue to contribute to significant advances in both areas.

## Author contributions

MP-V, AR, ER, ML, and OC-e-S conceived the Research Topic's idea and wrote the paper. All authors approved and corrected the final version of the manuscript.

## Funding

This work was supported by FCT UIDB/04501/2020—Base (MP-V and OC-e-S) and FEDER PT2020 01/45-SAICT/2021 PdC no: 1811255.

## Conflict of interest

The authors declare that the research was conducted in the absence of any commercial or financial relationships that could be construed as a potential conflict of interest.

## Publisher's note

All claims expressed in this article are solely those of the authors and do not necessarily represent those of their affiliated organizations, or those of the publisher, the editors and the reviewers. Any product that may be evaluated in this article, or claim that may be made by its manufacturer, is not guaranteed or endorsed by the publisher.

## Footnotes

^1^Food and Drug Administration (FDA). https://www.fda.gov/medical-devices/digital-health-center-excellence/what-digital-health.

